# Chiral Interface of Amyloid Beta (Aβ): Relevance to Protein Aging, Aggregation and Neurodegeneration

**DOI:** 10.3390/sym12040585

**Published:** 2020-04-07

**Authors:** Victor V. Dyakin, Thomas M. Wisniewski, Abel Lajtha

**Affiliations:** 1 Departmemts: Virtual Reality Perception Lab. (VV. Dyakin) and Center for Neurochemistry (A. Lajtha), The Nathan S. Kline Institute for Psychiatric Research (NKI), Orangeburg, NY 10962, USA;; 2 Departments of Neurology, Pathology and Psychiatry, Center for Cognitive Neurology, New York University School of Medicine, New York, NY 10016, USA;

**Keywords:** biochirality, post-translational modifications, protein folding, protein aggregation, spontaneous chemical reactions, neurodegeneration, non-equilibrium phase transitions

## Abstract

Biochirality is the subject of distinct branches of science, including biophysics, biochemistry, the stereochemistry of protein folding, neuroscience, brain functional laterality and bioinformatics. At the protein level, biochirality is closely associated with various post-translational modifications (PTMs) accompanied by the non-equilibrium phase transitions (PhTs ^NE^). PTMs ^NE^ support the dynamic balance of the prevalent chirality of enzymes and their substrates. The stereoselective nature of most biochemical reactions is evident in the enzymatic (Enz) and spontaneous (Sp) PTMs (PTMs ^Enz^ and PTMs ^Sp^) of proteins. Protein chirality, which embraces biophysics and biochemistry, is a subject of this review. In this broad field, we focus attention to the amyloid-beta (Aβ) peptide, known for its essential cellular functions and associations with neuropathology. The widely discussed amyloid cascade hypothesis (ACH) of Alzheimer’s disease (AD) states that disease pathogenesis is initiated by the oligomerization and subsequent aggregation of the Aβ peptide into plaques. The racemization-induced aggregation of protein and RNA have been extensively studied in the search for the contribution of spontaneous stochastic stereo-specific mechanisms that are common for both kinds of biomolecules. The failure of numerous Aβ drug-targeting therapies requires the reconsolidation of the ACH with the concept of PTMs ^Sp^. The progress in methods of chiral discrimination can help overcome previous limitations in the understanding of AD pathogenesis. The primary target of attention becomes the network of stereospecific PTMs that affect the aggregation of many pathogenic agents, including Aβ. Extensive recent experimental results describe the truncated, isomerized and racemized forms of Aβ and the interplay between enzymatic and PTMs ^Sp^. Currently, accumulated data suggest that non-enzymatic PTMs ^Sp^ occur in parallel to an existing metabolic network of enzymatic pathways, meaning that the presence and activity of enzymes does not prevent non-enzymatic reactions from occurring. PTMs ^Sp^ impact the functions of many proteins and peptides, including Aβ. This is in logical agreement with the silently accepted racemization hypothesis of protein aggregation (RHPA). Therefore, the ACH of AD should be complemented by the concept of PTMs ^Sp^ and RHPA.

## 1. Introduction

**Figure1.**
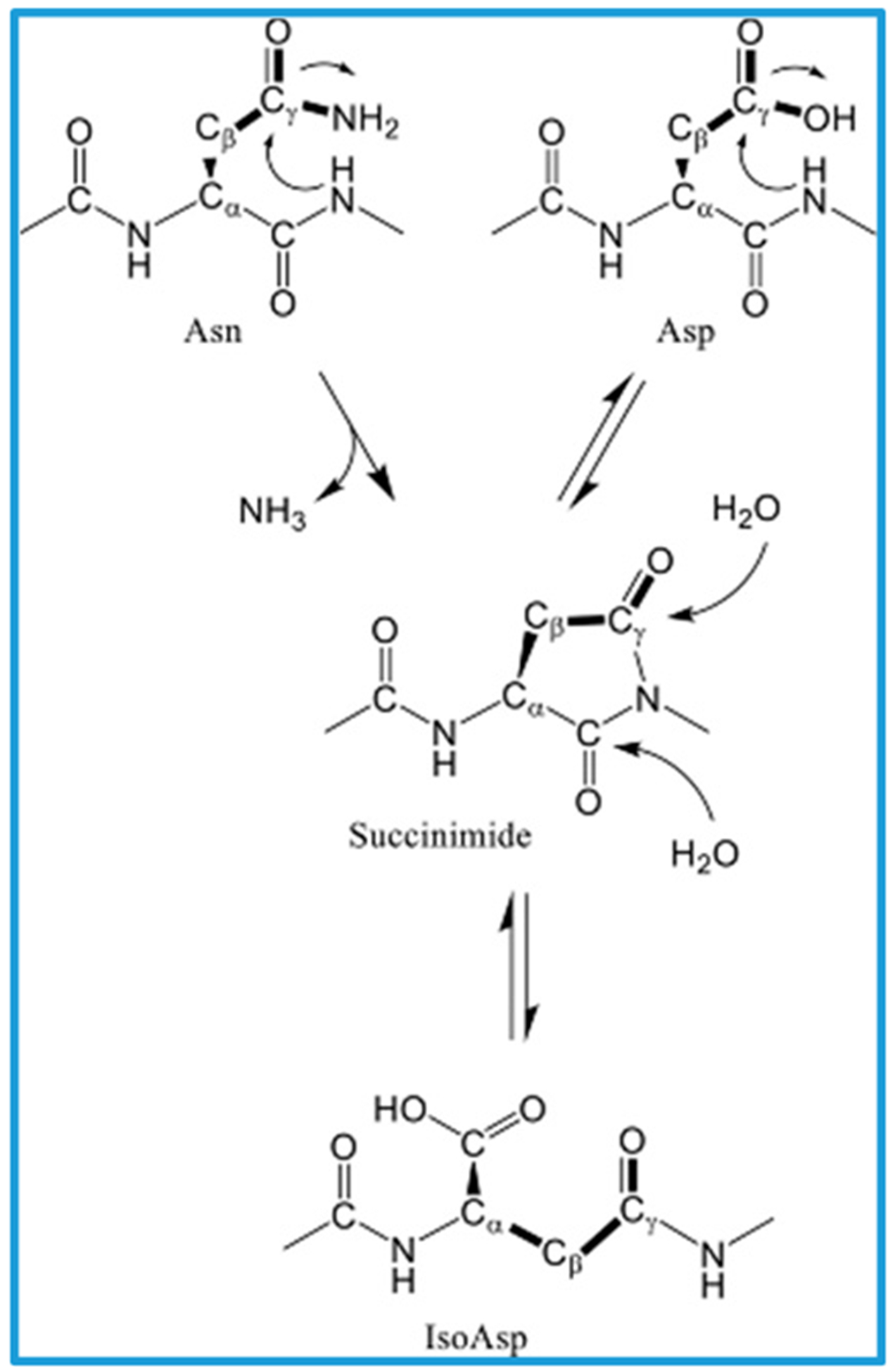
Spontaneous deamidation and isomerization of asparagine (Asn). Side-chain bonds of asparagine and aspartate are drawn as bold lines. Adopted from [4].

The amyloid cascade hypothesis (ACH) has played a crucial role in the understanding of the Alzheimer’s disease (AD) etiology and pathogenesis. The deposition of β-amyloid (Aβ) and neurofibrillary tangles (NFTs) traditionally served as the essential neuropathological features of AD. However, for many years, the attention to the stereochemistry of underlying spontaneous events was under-appreciated. Stereochemical errors in biomolecular structures, including proteins and peptides, have a dramatic impact on cell physiology [1]. The discovery of free D-aspartic acid (D-Asp) in rodents and man open a new window for understanding the mechanisms of protein synthesis and degradation [2]. Proteins, including glycoproteins, are the subjects of the reversible enzymatic (Enz) [3] and irreversible spontaneous (Sp) [4] post-translational modifications (PTM ^Enz^ and PTMs ^Sp^). (see Figure 1). All physiological and pathological forms of proteins are the consequence of PTMs. We are focusing on the aberrant forms of PTM, such as racemization ^Sp^ and isomerization ^Sp^. The relevance of the spontaneous modifications of amino acids (AAs) within peptides and long-lived proteins to protein aging, accumulation and pathologies is being increasingly recognized in the recent studies. Accordingly, the primarily biomarkers of aging and neurodegeneration are becoming the protein-cell-specific PTMs ^Sp^ of amino acids (AAs) [5–50].

## 2. Racemization of the Aβ

With the recognition of the fact that many proteins (Aβ, TAU, prion protein Prion (PrP), Huntingtin and α-synuclein) are the substrate of the aggregation-prone PTMs ^Sp^ [51], we are focusing, primarily, on the racemization of the Aβ. The amyloid precursor protein (APP) is one of the most studied proteins concerning pathological misfolding. The products of APP processing by α-, β- and γ-secretases range from 16 to 49 AAs.

Most studied amyloid beta (Aβ) peptides includes Aβ (1–16), Aβ (1–19), Aβ (20–34), Aβ (20–33), Aβ (20–40), Aβ (23–34), Aβ (34–40), Aβ (35–40), Aβ (1–35), Aβ (1–40) and Aβ (1–42) [5,52] are characterized by differential chain of PTMs and susceptibility to PTMs ^Sp^. PTMs of Aβ (1–42), the primary constituent of Aβ plaques in the AD brain, are extensively studied. Misfolding and aggregation of Aβ peptides is the convincing example of a link between the perturbations of the molecular chirality, deteriorated enzyme-substrate recognition, abnormal cell signaling (including neurotransmission) and cognitive dysfunction [3]. The spontaneous aggregation of Aβ peptides into amyloid plaques and in the walls of the cerebral vasculature is the unresolved issue of Alzheimer’s disease (AD)-amyloid conundrum [6]. It is a common assumption that PTMs ^Sp^ can significantly alter the structure of the original polypeptide chain. The AAs that most frequently undergo racemization ^Sp^ and isomerization ^Sp^ in human proteins are aspartate (Asp), asparagine (Asn), glutamate (Glu), glutamine (Glu), serine (Ser), alanine (Ala) and proline [7]. For Aβ peptides, racemization-prone are found two non-essential AAs: serine (Ser) and aspartate (Asp) (Aβ−42 contains two Ser and three Asp residues (see Table 1)). For Asp, the mechanism acceleration of racemization ^Sp^ (about 10 ^5^) is associated with the specific succinimide intermediates [8–10]. Both D-Ser and D-Asp play a crucial role in N-methyl-d-aspartate (NMDA) receptor-mediated neurotransmission. D-Ser26-Aβ1–40 possesses a strong tendency to form fibrils [11]. AD patients have increased brain D-Ser levels [12]. This fact agrees with the activated spontaneous racemization (Rs^Sp^) of Ser residue in Aβ, with an elevated level of D-Ser in amyloid plaques, impairment of the NMDA neurotransmission, memory loss and cognitive dysfunction. Racemization and isomerization of Asp are the most common types of non-enzymatic covalent modification that leads to an accumulation of aging proteins in numerous human tissues [13]. Asp-1, Asp-7 and Asp-23 of Aβ are crucial in the control of Aβ aging and aggregation [5].

Residues Asp-1 and Asp-7 of Aβ in amyloid plaques are a mixture of L-, D-, L-iso- and D-iso-aspartate [14]. D-Asp-7 enhances the aggregation process by shifting the equilibrium of Aβ from the soluble to the insoluble form [15]. Therefore, the set of PTMs ^Sp^, including racemization ^Sp^ and isomerization ^Sp^, is an efficient modifier of Aβ metabolism. In 2011, Kumar promoted the hypothesis that enzymatic phosphorylation of Aβ triggers the formation of toxic aggregates [16], which has been confirmed by later studies [3]. In 1994, Szendrei discovered that spontaneous isomerization of Asp affects the conformations of synthetic peptides [17]. However, the role of the PTMs ^Sp^ in Aβ aggregation and neurotoxicity remains in the shadow. Consequently, many structural details of misfolded Aβ have remained elusive for a long time [7,18]. This short review provides a summary of information regarding events of PTMs ^Sp^ in Aβ. The heterogeneity of Aβ proteolytic forms in AD brain is represented by at least 26 unique peptides, characterized by various N- and C-terminal truncations. The N- and C-terminal truncated fragments (in contrast to canonical Aβ) are allowing to distinguish between the soluble and insoluble aggregates. The N-terminal truncations are predominating in the insoluble material and C- terminal truncations segregating in the soluble aggregates [19,20]. Aβ peptides exhibit a high sensitivity of the secondary structure and fibril morphologies to the chirality of ligands [21] and enzymes of PTMs. Only for a small part of Aβ isoforms exist information regarding the pathways of PTMs ^Sp^.

Currently, available data for Aβ42 peptides are summarized in Tables 1–3. The data in Tables 2 and 3 demonstrate two essential facts: first, the coincidence of phosphorylation and spontaneous racemization/isomerization (enzymatic phosphorylation can be accompanied by the enzyme-driven or spontaneous racemization) events at the Ser-8, Ser-26 (Ser-26 residue is located within the turn region of Aβ), and Asp23 residues, second, currently available information covering the PTMs ^Sp^ of Aβ is limited only to 4 from the 16 types of AAs, which means that much remains to be explored. Most recent attempts to overcome previous limitations of the ACH are concentrated on many additional essential aspects of metabolism, contributing to progress in understanding [22–42]. However, most them, do not pay enough attention to the stereochemistry of the PTM in general and the impact of spontaneous racemization (^Sp^) on Aβ assembly, aggregation and functions. At the same time, the progress in methods of chiral discrimination has produced new, stereochemistry-oriented, experimental results regarding aberrant PTMs ^Sp^. Growing evidence suggests that proteins undergo several unusual, previously unknown PTMs associates with the interplay between physiological protein modification, spontaneous aging-associated molecular processes [7,10,11,13], stress conditions [23,24], accidental co-localization of the enzyme and substrate or PTMs ^Sp^. For Aβ, Asp and Ser are known as the most racemization prone residues. For the illustration purpose, we provide several of many existing molecular pathways where the racemization ^Sp^ of Ser can be critical.

First, the pathological role of mitochondrial enzymes Ser proteases (SerPs) is attributed to neurodegenerative disorders such as AD and Parkinson’s and disease [25–27]. The HtrA (high-temperature requirement) family represents a class of oligomeric SerPs [25]. Its members are classified by presence (in its AAs sequence) a catalytic triad contains His, Asp and Ser residues known as racemization prone.

Second, chaperone signaling complexes in AD involve a wide range of heat shock proteins (Hsp), including Hsp27, that are ingaged in protection against Aβ aggregation and toxicity [28]. Human Hsp27 is phosphorylated at three Ser residues (Ser15, Ser78 and Ser82), were Ser-78 and Ser-82 are the major phosphorylation sites [29,30]. It is evident that due to the stereo-specificity activity of both protein types (SerPs and Hsps) racemization ^Sp^ of Ser residues in each of them will contribute to the aberrant processing of substrates, including Aβ, inducing the cascade of aggregation, accompanied by neurodegeneration. The aggregation of protein and peptide indicates the decrease in the turnover rate. Accordingly, the previously short half-life-proteins are changing in the direction toward the long-lived one. Lowering turnover rate (i.e., protein aging) makes proteins the subject of the time-dependent PTMs ^Sp^, including oxidation, nitration, glycation, isomerization and racemization [7]. The set of PTMs ^Sp^ and its effect on protein polymerization both are substrate specific. Tyrosine (Tyr) nitration, for example, significantly decreased the aggregation of Aβ1–40. [43].

## 3. Conclusions

In the manuscript, we assess the previous and current experimental results acquired in the specific areas of chiral proteomics—Aβ folding—from a broad perspective. For this purpose, we addressed the basic, fundamental and widely recognized facts and theories underlying the stereochemistry of Aβ. Due to progress in multidisciplinary fields, the view of the origin of biologic non-equilibrium chirality evolves from the physico-chemical nature of enantioselective autocatalytic reaction networks to a process that play an essential role in the pathogenesis of AD [44]. The phenomena of biochirality embrace two undivided branches of science biophysics and biochemistry. In 1990th, the nature of living organisms was associated with the absolute homochirality [50]. With the discovery of D-AAs in living organisms and the process of enzymic racemization, the concept of homochirality was replaced by the notion of prevalent chirality.

In the language of entropy, the transfer of protein/solvent system from the state of low-entropy (racemic mixture) to the high-entropy state (homochirality) is the order-disorder type transitions.

In terms of thermodynamics, this is the transition from the non-equilibrium to the equilibrium state. Accordingly, the enzymic PTMs, from a biophysical perspective, is the set of physiological non-equilibrium phase transitions. Enzymic racemization is an essential and necessary source of D-AAs in organisms. In contrast, the spontaneous racemization, as an aberrant PTMs, is the window for the irreversible transfer from non-equilibrium to equilibrium conditions.

In the words of proteomics, irreversible racemization is the conformation of protein from functional (physiological) to the dis-functional (inert or toxic) state of protein solvent, aggregates and depositions. The universal significance of the symmetry constraints is evident from the viral to the human proteome. The biologic significance of racemization-induced protein aggregation for the neuropathogenesis of AD was experimentally demonstrated as early as 1994 1994 [45]. Currently accumulated data about PTMs of many proteins and peptides, including Aβ are coherent with the silently accepted racemization hypothesis of protein aggregation (RHPA). Therefore, after “three decades of struggles, ACH [46] of the neurodegeneration should be complemented by the concept of PTMs ^*Sp*^ and non-equilibrium phase transitions (PhTs ^NE^) [47–50].

## Figures and Tables

**Table 1.** The frequency (f) of the AAs appearance in Aβ (1–42).

The Frequency (f) of the AAs Appearance in A-beta (1–42)					
f	Amino Acids				
6	Gly	Val			
4	Ala				
3	**Asp**	Glu	Phe	His	Ile
2	Lys	Leu	**Ser**		
1	Met	Asn	Arg	Gln	Tyr

**Table 2.** Coincidence of enzymatic and spontaneous PTMs at Ser and Asp residues of Aβ in the neurodegenerative amyloid aggregates.

Peptide	Disease	Residue	PTMs		
			Rcm.	Ism.	Ph.
			Spontaneous		Enzymic
		**Ser-8**	[24, 35]		[3, 40, 41, 42]
**A-β (40–42)**	AD	**Ser-26**	[13, 35, 36]		[43. 44]
		**Asp-23**	[35, 37, 38]		[40]
**A-β (20–34)**		**Asp-23**		[39]	

Post-translational modification (PTMs): Spontaneous (Sp) and Enzymic (Enz), Racemization (Rcm). Isomerization (Ism). Phosphorylation (Ph).

**Table 3.** Coincidence of phosphorylation and racemization at Ser and Asp residues of Aβ (1–42).

A-Beta (1–12)																																											
**N-Terminal**	1	2	3	4	5	6	7	8	9	10	11	12	13	14	15	16	17	18	19	20	21	22	23	24	25	26	27	28	29	30	31	32	33	34	35	36	37	38	39	40	41	42	**C-Terminal**
	Asp	Ala	Glu	Phe	Arg	His	Asp	**Ser**	Gly	Tyr	Glu	Val	His	His	Gln	Lys	Leu	Val	Phe	Phe	Ala	Glu	**Asp**	Val	Gly	**Ser**	Asn	Lys	Gly	Ala	Ile	Ile	Gly	Leu	Met	Val	Gly	Gly	Val	Val	Ile	Ala	
	D	A	E	F	R	H	D	**S**	G	Y	E	V	H	H	Q	K	L	V	F	F	A	E	**D**	V	G	**S**	N	K	G	A	I	I	G	L	M	V	G	G	V	V	I	A	
								*																		*																	
																							**																				
								***															***			***																	

PTM: Enzymic (***) and Spontaneous (Racemization *, Izomerization **).

## Abbreviations

ACH: Amyloid cascade hypothesis
PTMs: post-translational modifications
PTMs: Enz enzymic PTMs
PTMs: Sp spontaneous PTMs
PhTs: NE non-equilibrium phase transitions
Aβ: amyloid beta
RHPA: racemization hypothesis of protein aggregation
Ala: Alanine
Asn: Asparagine
Ser: Serine
Asp: aspartic acid
Glu: Glutamate
Ile: Isoleucine
Tyr: Tyrosine
Pro: Proline
